# Genetic Tracing of Clonal Expansion and Progression of Pancreatic Ductal Adenocarcinoma: A Case Report and Multi-Region Sequencing Analysis

**DOI:** 10.3389/fonc.2020.00728

**Published:** 2020-06-02

**Authors:** Shion Tachibana, Yusuke Mizukami, Yusuke Ono, Yuya Sugiyama, Tetsuhiro Okada, Arisa Kitazaki, Junpei Sasajima, Motoya Tominaga, Jun Sakamoto, Keisuke Kimura, Yuko Omori, Toru Furukawa, Taichi Kimura, Shinya Tanaka, Kazuo Nagashima, Hidenori Karasaki, Tomoyuki Ohta, Toshikatsu Okumura

**Affiliations:** ^1^Institute of Biomedical Research, Sapporo Higashi Tokushukai Hospital, Sapporo, Japan; ^2^Center for Gastroenterology, Sapporo Higashi Tokushukai Hospital, Sapporo, Japan; ^3^Department of Medicine, Asahikawa Medical University, Asahikawa, Japan; ^4^Department of Investigative Pathology, Tohoku University Graduate School of Medicine, Sendai, Japan; ^5^Department of Pathology, National Hospital Organization Hokkaido Medical Center, Sapporo, Japan; ^6^Department of Cancer Pathology, Hokkaido University Graduate School of Medicine, Sapporo, Japan

**Keywords:** pancreatic cancer, premalignant field defect, clonal evolution, multi-region sequencing, *KRAS*

## Abstract

Pancreatobiliary tumors frequently contain multiple malignant and precancerous lesions; however, the origin of the driver mutations and the mechanisms that underlie the generation of distinct clones within an organ field remain unclear. Herein, we describe a 76-year-old male suffering from moderately differentiated adenocarcinomas of the pancreas that primarily involved the distal bile duct and multiple “dispersing” invasive lesions in the pancreatic head. The patient underwent pylorus-preserving pancreaticoduodenectomy with superior mesenteric vein resection, and targeted sequencing of 18 genes associated with pancreatic tumorigenesis and immunohistochemical analysis of RNF43 and ARID1A were performed on each tumor compartment, including the invasive and non-invasive areas. Multi-region sequencing revealed shared *KRAS* and *TGFBR1* mutations in all invasive foci, including those involving the distal bile duct. Distinct *KRAS* variants were found to be present in other non-continuous and non-invasive lesions in the pancreas. Intraductal lesions with *KRAS* G12D and *RNF43* V50R mutations were evident in the main pancreatic duct. This appeared to be a founder clone, given that the mutation profile was common to the invasive foci as well as the additional high-grade dysplasia harboring *ARID1A* mutations, thereby suggesting a clonal branch-off during tumor evolution. In addition, we also observed independent intraductal papillary mucinous neoplasms with *KRAS* G12V and *GNAS* R201H mutations. Our theory, learned from this patient, was that lesions skipped dissemination and wide-spread movement potentially through the pancreatic ductal system as a process of pancreatic cancer development.

## Introduction

A subset of patients with pancreatobiliary tumors is prone to developing multiple precancerous and malignant lesions, similar to cancers that affect other organs, which is a common phenomenon in a variety of malignancies referred to as field carcinogenesis (1, 2). Despite their close anatomical locations and similarity in early developmental pathways, tumors that develop in the pancreatobiliary systems may exhibit organ-specific evolutionary trajectories during carcinogenesis. For instance, the presence of multiple lesions is ubiquitously observed in intraductal papillary mucinous neoplasms (IPMNs) of the pancreas and is associated with an increased risk of pancreatic cancer (3, 4), whereas multi-focal biliary tract cancer is considerably rarer (5–7).

Despite the well-established model of tumor evolution from benign to malignant lesions via the acquisition of a series of mutations over time (8), the specific time course of the occurrence of these driver mutations and the mechanism underlying the generation of distinct clones within an organ remain unclear (9). The step-wise progression model postulates a scenario in which multi-focal tumors originate from a single clone that has acquired a common driver mutation. In addition to such linear tumor evolution, multiple independent early clones can emerge during aging in general populations in association with lifestyle risks and in high-risk individuals with genetic variants. However, evaluating tumor evolution by collecting longitudinal human samples presents considerable challenges (10).

A recent study based on whole-exome sequencing and phylogenetic analysis has indicated that discrete pancreatic precursors in a single pancreas often represent a unique neoplasm that has colonized through the ductal system, subsequently undergoing spatial and genetic divergence over time (11). Here, we present details of a case of pancreatic cancer in which multiple invasive foci were distributed in the head of the pancreas with a premalignant field defect, which was potentially related to IPMN. By targeting the main pancreatic ductal adenocarcinoma (PDA)-related genes using multi-regional paired tumor and germline DNA sequencing, we were able to establish the evolutionary history of the emergence of ancestral tumor cells and associated subclones, one of which led to the development of discontinuous PDAs. On the basis of such a “snapshot” analysis, it is possible to elucidate the origins and the process of the clonal evolution of the precursor lesion toward invasive cancer, offering further insights into the mechanisms of heterogeneous malignancy with respect to carcinogenesis and its diagnosis.

## Methods

### Ethical Approval

The genetic analysis performed in this study was approved by the Institutional Review Board of the Tokushukai Group Ethical Committee on Human Research (#TGE00357-012), and written informed consent was obtained from the patient for publication of the case details.

### Histological Evaluation

The whole resected pancreatectomy specimen was fixed in 10% neutral-buffered formalin solution for 48 h and transferred to saline. Tissues were sliced at 5-mm intervals, embedded in paraffin, deparaffinized, rehydrated, and stained with hematoxylin and eosin (H&E). Exhaustive tumor mapping of the specimens was performed for molecular tracing based on precise pathological navigation (4). The entire resected specimens, sliced at 5-mm intervals, were employed. All histological sections of the resected pancreas were carefully examined by three pathologists who assessed the distribution of invasive and non-invasive tumor compartments. Furthermore, dysplasia, presumably containing either a pancreatic intraepithelial neoplasia (PanIN) or an incipient IPMN, were mapped on the resected specimens (12). Distances between lesions and distribution patterns of the associated neoplastic foci were assessed (i.e., cancerization of the duct or discontinuous skip dissemination).

Immunohistochemical staining of the proteins RNF43 and ARID1A was performed on the invasive tumor and dysplasia using the following antibodies: anti- RNF43 (HPA008079, 1:100; Sigma-Aldrich, St Louis, MO, USA) and anti-ARID1A (clone EPR13501, 1:1,000; Abcam, Cambridge, UK). Loss of expression was assessed as aberrant protein expression (4).

### DNA Isolation

Genetic analysis was performed using formalin-fixed, paraffin-embedded tissue specimens. Genomic DNA was purified and isolated from invasive and non-invasive tumor compartments, as well as from microscopic dysplastic lesions in the normal-looking pancreas and duodenum tissue using a GeneRead DNA FFPE Kit (Qiagen, Hilden, Germany).

### Targeted Amplicon Sequencing

A PDA/IPMN-associated gene panel (Ion AmpliSeqTM Custom DNA Panel) was designed using Ion AmpliSeq Designer 3.6 to analyze the coding DNA sequences + 25 bp from the intronic flanking regions of 18 genes, namely, *KRAS, TP53, SMAD4, CDKN2A, GNAS, RNF43, PIK3CA, STK11, BRAF, TGFBR1, TGFBR2, MAP2K4, ARID1A, KDM6A, SF3B1, RBM10, IDH1*, and *CTNNB1*, as previously described (4). In brief, DNA (10–60 ng) was amplified using the panel, and a sequencing library was prepared. Sequencing and data analyses were performed using an Ion PGM System (Thermo Fisher Scientific, Waltham, MA, USA). Sequenced reads were de-multiplexed, quality-filtered, and aligned to the human reference genome (GRCh37) using the Torrent Suite software package (ver. 5.0.4; Thermo Fisher Scientific). For variants containing novel exonic, non-synonymous, and frameshift insertion/deletions as well as intronic splice variants, the COSMIC (http://cancer.sanger.ac.uk/cosmic) and ClinVar databases (https://www.ncbi.nlm.nih.gov/clinvar/) were used for their classification as either pathogenic or variants of unknown significance.

## Results

**Figure 1:**
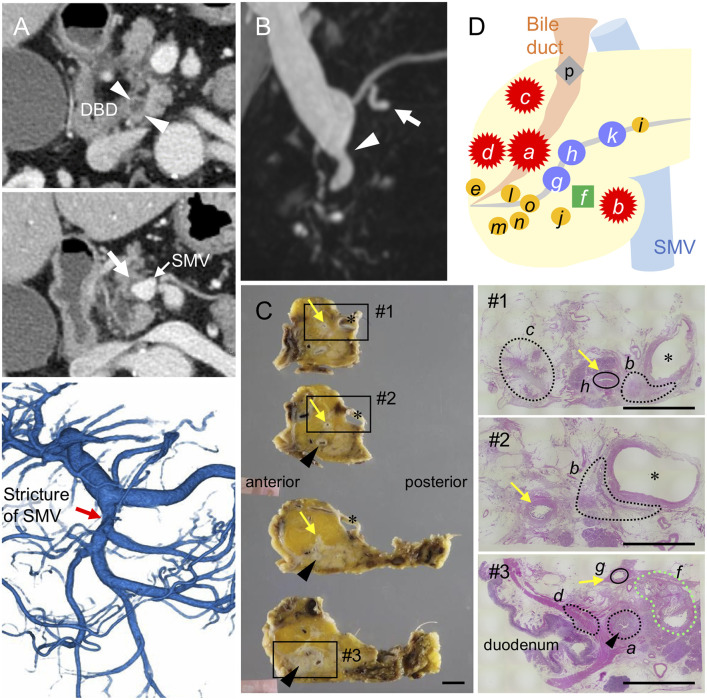
Distribution of invasive and intraductal lesions in the pancreas. Pre-operative images showing a stricture in the distal bile duct (DBD) (arrowheads in **A,B**) with no apparent tumors detected in the surrounding tissues. Note the abnormal density in the pancreatic parenchyma surrounding the superior mesenteric vein (SMV) (white arrow in **A**) visualized by an axial slice of the contrast-enhanced computed tomography, stricture of the SMV (red arrow in **A**) by 3D-reconstruction of the portal phase, and small dilatations in the branch duct (white arrow in **B**) detected by magnetic resonance imaging **(B)**. **(C)** Resected specimens and corresponding hematoxylin and eosin images showing the distribution of each invasive cancer and intraductal neoplasm. Areas marked by black and green dashed lines indicate the invasive compartments and intraductal papillary mucinous neoplasm (IPMN), respectively. Note that the areas marked by black circle were high-grade dysplasias found in the main pancreatic duct (yellow arrows); asterisk, SMV. Black arrowheads indicate common bile duct (CBD). Scale bars, 1 cm. **(D)** Schema of tumor compartments from which DNA was isolated for genotyping: red star, invasive carcinoma; blue circle, high-grade pancreatic intraepithelial neoplasia (PanIN); yellow circle, low-grade PanIN; green square, IPMN; gray diamond shape, low-grade atypia.

A 76-year-old man was referred to our hospital because of jaundice. The patient had neither a medical history of pancreatobiliary diseases nor a family history of cancer. He was a heavy smoker (Brinkman Index: 900) with no history of heavy drinking. The laboratory data showed an elevation of total bilirubin (5.6 mg/dL) and normal levels of the pancreatic enzymes, carbohydrate antigen 19-9 (CA19-9), and IgG4. Contrast-enhanced computed tomography revealed stenosis in the distal bile duct and a low-density area in the pancreatic parenchyma surrounding the superior mesenteric vein (SMV), separate from the bile duct (Figure 1A). Magnetic resonance cholangiopancreatography revealed dilatations of the pancreatic branch ducts around the stricture of the distal bile duct; however, no pancreatic or bile duct junction abnormalities were detected (Figure 1B). Although a tissue specimen from the bile duct revealed an adenocarcinoma, no histological evidence was obtained from the pancreatic duct tissue.

The patient underwent pylorus-preserving pancreaticoduodenectomy with SMV resection. Histological analysis of the surgical specimens revealed a moderately differentiated adenocarcinoma in the whole circumference of the distal bile duct with infiltration into the duodenum and surrounding adipose tissue. A similar invasive compartment was also found in the pancreatic parenchyma surrounding the SMV, and a gastric-type IPMN was located close to the distal bile duct stenosis (Figure 1C). In addition to the macroscopically identified lesions, histological examination of the apparent normal-looking pancreas showed high-grade PanIN in the main pancreatic duct and scattered low-grade branch duct lesions (Figure 1D). Lympho-vascular invasion, peri-neural invasion, and regional lymph node metastasis were histologically observed, but no distant metastasis was evident (UICC 8th; T2 N1M0, Stage IIB).

**Figure 2:**
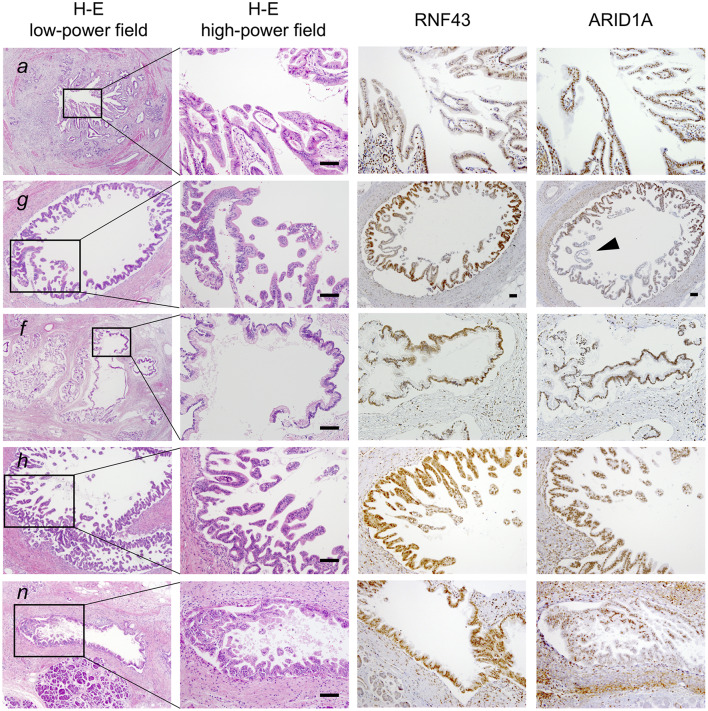
Histological evaluation of multiple tumor foci in the resected pancreas. Pathological findings of representative tumor compartments utilized for multi-region sequencing; hematoxylin and eosin staining (left panel; low- and high-powered view) and images of RNF43 and ARID1A immunohistochemistry (right panels; high-powered view with same magnification as H&E) are shown: *a*, pancreatic ductal adenocarcinoma (PDA) in the distal bile duct; *g*, high-grade pancreatic intraepithelial neoplasia (PanIN) in the main pancreatic duct (MPD); *f*, low-grade intraductal papillary mucinous neoplasm (IPMN) in a branch duct; *h*, high-grade PanIN in the MPD; *n*, low-grade PanIN in the MPD. Note partial loss of expression of ARID1A as indicated by the arrowhead. Scale bars, 100 μm.

To clarify whether the histologically discontinuous infiltrating lesions distributed in the pancreas originated from a common precursor or multiple independent tumors, we profiled their mutations via amplicon resequencing targeting of common PDA-associated genes (1). Several low-grade PanINs with various types of *KRAS* mutations (G12D, G12V, and G12R) were distributed in the resected pancreatic specimens (Table 1, Figure 2, Supplementary Table 1). High-grade PanINs harboring *KRAS* G12D and *RNF43* V50R mutations were found in the main pancreatic duct (MPD), which were distinct from the mutations in the low-grade PanIN, despite the common *KRAS* variant signature. All invasive compartments, including the distal bile duct lesions, were clonally related to the high-grade PanINs with *KRAS* G12D and *RNF43* V50R mutations in the MPD, as the apparent founder tumor, and then acquired additional mutations in *TGFBR1* (E499X) and *RBM10* (R765X) (Table 1, Figure 2). Moreover, another high-grade PanIN in the MPD was found to be related to the founder lesion marked by *KRAS* G12D and *RNF43* V50R mutations but had distinct promotive mutations in the *ARID1A* (P650fs and R1335X) and *RBM10* (S288X) genes, which emerged after the development of the earliest tumor clone with a minimum set of oncogenes. Since the pathogenesis of the tumor suppressor variants found in these tumor compartments is ambiguous thus far, immunohistochemical studies were performed on ARID1A and *RNF43*, in which specific antibodies are commercially available and validated (4). The analysis revealed the partial loss of ARID1A expression that is suggestive of loss-of-function mutations, whereas the expression of *RNF43* was preserved in tumor components with *RNF43* V50R (Figure 2). In addition to *KRAS* G12V, a *GNAS* mutation was detected in the gastric-type IPMN located adjacent to the distal bile duct stenosis.

**Table 1:** Mutation signatures of each tumor compartment by multi-region sequencing targeting common PDA-associated genes.

	**Region**	**Histology**	***KRAS***	***GNAS***	***RNF43***	***TGFBR1***	***RBM10***	***ARID1A***
a	Distal bile duct	PDA	G12D		V50R	E499X	R765X	
b	Peri SMV	PDA	G12D		V50R	E499X	R765X	
c	Adipose tissue	PDA	G12D		V50R	E499X	R765X	
d	Duodenal wall	PDA	G12D		V50R	E499X	R765X	
e	BD in Ph	Low-grade PanIN	G12D					
f	BD in Ph	Low-grade IPMN	G12V	R201H				
g	MPD in Ph	High-grade PanIN	G12D		V50R		S288X	P650fs, R1335X
h	MPD in Ph	High-grade PanIN	G12D		V50R			
i	MPD in Pb	Low-grade PanIN	G12V					
j	BD in Ph	Low-grade PanIN	G12R					
k	MPD in Ph	High-grade PanIN	G12D		V50R			
l	BD in Ph	Low-grade PanIN	G12D					
m	BD in Ph	Low-grade PanIN	G12D					
n	BD in Ph	Low-grade PanIN	G12D					
o	MPD in Ph	Low-grade PanIN	G12V					
p	Middle bile duct	Low-grade atypia	WT					

*BD, bile duct; IPMN, intraductal papillary mucinous neoplasms; MPD, main pancreatic duct; PanIN, pancreatic intraepithelial neoplasia; Pb, pancreatic body; PDA, pancreatic ductal adenocarcinoma; Ph, pancreatic head; SMV, superior mesenteric vein*.

**Figure 3:**
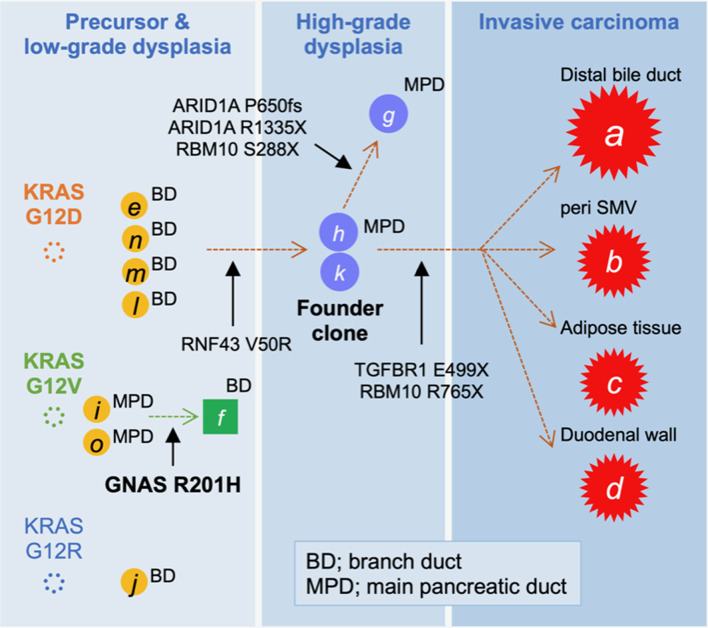
Tumor evolution map displaying the accumulation of mutations. The high-grade pancreatic intraepithelial neoplasia (PanIN) found in the main pancreatic duct (MPD), marked by “*h*” and “*k*,” was considered to be the founder lesion that progressed to an invasive carcinoma (**A–D**; red stars) by acquiring a TGFBR1 mutation, whereas the branched subclone with ARID1A mutations remained as intraductal lesions.

On the basis of the disease distribution and mutation accumulation profile (Figure 3), we concluded that the precursor harboring a *KRAS* G12D mutation had acquired an *RNF43* V50R mutation to establish a “founder clone” that subsequently acquired various additional mutation types, thereby enabling it to spread throughout the pancreas. Among the subclones, the tumor region with *TGFBR1*, but not ARID1A, mutations developed discontinuous infiltrating lesions in a skipping manner within the pancreas. Given the genetic analyses, adenocarcinoma of the pancreas was more likely to develop, and therefore, Tegafur/gimeracil/oteracil (S-1) was selected for adjuvant chemotherapy. At 1 year after the resection, the patient showed no signs of recurrence.

## Discussion

Distinguishing between ductal adenocarcinoma of the pancreatic head and distal cholangiocarcinoma can occasionally present a challenge due to their common embryologic origin, close anatomical location, and several phenotypic analogies (13). In addition to complicating decision-making or management for such patients, these complexities make it difficult to determine the cell-of-origin of these neoplasms and their mode of expansion (14, 15). Activating mutations in *KRAS* occur almost ubiquitously in PDA and its putative precursor lesions (3), whereas the frequency of such mutations is moderate in biliary tract cancer, including ampullary cancer (16, 17). Despite the independence of these tumor entities based on genetics, the origins of these tumors may not have been appropriately dissected in previous studies. Here, using small-scale mutation profiling based on targeted sequencing, we successfully traced the evolution of the cancer and could identify the site of origin of the precursors in the pancreas that had progressed to an invasive carcinoma.

Among multi-centric tumors designated by distinct *KRAS* variants, all invasive compartments in the distal bile duct, pancreas, SMV surrounding, duodenum, and adipose tissue, despite being anatomically dispersed, were considered to be a series of lesions, given their common mutations in *KRAS, TGFBR1, RBM10*, and *RNF43*. Detailed sequencing, including that of the non-invasive compartment of the macroscopically normal-looking pancreas, revealed tumor regions, including high-grade PanINs in the MPD, which was histologically evident, with a minimum set of mutations (*KRAS* G12D and RNF43 V50R). This is suggestive of the founder clones preceding the invasive foci. If these non-invasive lesions had originated from tumors developed in the bile duct, we would not be able to explain the loss-of-*TGFBR1* E499X and *RBM10* R765X mutations. Therefore, we have excluded the possibility of the bile duct as the point of origin for the tumor. The absence of decreased RNF43 expression indicates that the *RNF43* V50R variant may be a passenger rather than pathogenic; the variant was useful to trace the clonal expansion from the potent founder lesion. Furthermore, we were unable to demonstrate the histological continuity of tumor compartments that were genetically associated. Therefore, it is more reasonable to assume that following the development of the founder tumor cells, a seeded cell moved toward a separate site of the pancreatic duct, rather than a scenario in which each lesion emerged independently. Hence, the key observations included identification of the intraductal lesions, which served as founder lesions in the main pancreatic duct with a minimum set of mutations shared by all invasive tumors. These compartments were separated by intervening pathological sections containing no histological or genetic evidence of tumors. There were significant distances between the invasive compartments (100–1,000 μm) with additional *TGFBR1* mutation. However, the precise measurement of the distances among the tumor compartment was challenging since the tissues were sliced at a 5-mm interval. Detailed assessment with comparative analysis of tissue reconstruction algorithms for 3D histology in combination with genetics will be needed to fully confirm the geographical extension of the founder clone. Interestingly, given the loss of the tumor suppressor ARID1A protein expression found in part of HG-PanINs located at MPD, *ARID1A* P650fs, and R1335X variants appeared to direct the founder lesion with *KRAS* G12D and *RNF43* V50R to branch a subclone distinct from the invasive lesions. Furthermore, to confidently exclude the possibility of tumor cell extension via the lymphatic-vascular system, sequencing of the foci is necessary; however, given the limited number of tumor cells, this was not possible in the current case study.

Successful identification of a founder clone in cases such as the one described herein may provide new insights into clonal evolution, and thereby enable us to gain a better understanding of how an indolent precursor with highly invasive and metastatic properties progresses to overt disease. Since all invasive components identified in the present study had common unique promotive mutations in the *TGFBR1* and *RBM10* genes, and the other lesion originated from a branched clone harboring *ARID1A* mutation, we determined that a subclone in the founder lesion acquired a *TGFBR1* mutation and then dispersed through the branches of the pancreatic duct in order to colonize and eventually invade different anatomical sites within and around the pancreas. Such estimates of accumulated spatial and genetic divergence over time obtained using multi-region sequencing in a tissue “snapshot” are consistent with the recently proposed linear progression model of PDA (11). Recently, in an elegant study targeting precancerous lesions, a temporal shift in the evolutionary principle was demonstrated to cause intratumor heterogeneity in colorectal cancer (18). Such efforts, in combination with the findings of the present study, will contribute to identifying the genetic processes underlying tumor evolutionary trajectories (4).

Among the non-invasive lesions found in the pancreas, incipient IPMNs with *KRAS* G12V and *GNAS* R201H mutations were observed near the high-grade PanINs in the MPD. The presence of such multi-centric tumor development is a common feature of IPMN (4); however, a premalignant field defect can also be generated in a subset of classic PDA without typical concurrent IPMN. Although we did not detect the sizable cystic lesion in the pancreas prior to surgery, minute dilatations in the branch ducts were visualized by magnetic resonance cholangiography. Thus, careful evaluation of imaging findings is required for comparison with histological and genetic assessments using resected specimens. Notably, small cystic lesions, as well as dilatation in the branch ducts, have frequently been observed in individuals with a higher risk of developing PDA (19–22). Accordingly, fine mapping using resected specimens, even in cases of sporadic pancreatic cancer, may provide significant insights for the development of novel diagnostic strategies. Pathological assessment of frozen sections for confirmation of the negative surgical margin is a standard procedure during surgical intervention; however, the findings of the current study, as well as the current lack of consensus regarding the significance of the surgical margin for prediction of local recurrence, suggest that there is a requirement for new tools that provide accurate information regarding the range of tumor spread. In addition to the potent multi-centric occurrence of early neoplastic clones, we may need to assess the potential of metachronous tumor development in the residual pancreas. As the current imaging technologies are incapable of visualizing such minute nests of tumors, genetic examination of the pancreatic juice following resection may be an alternative to cytological assessment, provided if rapid onsite genetic testing could be performed.

## Conclusions

Herein, we report a case characterized by a series of *KRAS*-driven precursors and associated PDA, which colonized and interspersed via the pancreatic ductal system. A detailed assessment of the entire tissue of the resected pancreatic specimen enabled us to elucidate the evolutionary trajectory of pancreatic cancer development. This case illustrates that such “snapshot” analyses with a thorough follow-up of time course changes in the residual pancreas can provide a better understanding of the evolutionary history of pancreatic cancer. The discontinuous skip dissemination from the founder clones could be more generally observed during pancreatic tumorigenesis, and this mode of progression will need to be carefully interpreted to estimate tumor recurrence following surgical intervention.

## Data Availability Statement

The original contributions presented in the study are included in the article/Supplementary Material, further inquiries can be directed to the corresponding author/s.

## Ethics Statement

Genetic analysis was approved by the Institutional Review Board of the Tokushukai Group Ethical Committee on Human Research (#TGE00357-012). The patients/participants provided their written informed consent to participate in this study and for publication of the case details.

## Author Contributions

STac, HK, and YM: design of the study. STac, YOn, and YM: drafting of the manuscript. YOm, TF, TK, STan, and KN: histological analysis. YOn, AK, HK, and YM: tumor sequencing and data interpretation. YS, TOka, JSas, MT, JSak, KK, TOh, and HK: coordination of clinical care and specimen procurement and analysis. TOku: study supervision. All authors have read and approved the manuscript.

## Conflict of Interest

YM and YOn have received funding from Hitachi High-Tech Corporation. The remaining authors declare that the research was conducted in the absence of any commercial or financial relationships that could be construed as a potential conflict of interest.

## Abbreviations

PDA: pancreatic ductal adenocarcinoma
IPMN: intraductal papillary mucinous neoplasm
SMV: superior mesenteric vein
MPD: main pancreatic duct.
